# Emerging Roles of the MICOS Complex in Cristae Dynamics and Biogenesis

**DOI:** 10.3390/biology10070600

**Published:** 2021-06-29

**Authors:** Ruchika Anand, Andreas S. Reichert, Arun Kumar Kondadi

**Affiliations:** Institute of Biochemistry and Molecular Biology I, Medical Faculty and University Hospital Düsseldorf, Heinrich-Heine University Düsseldorf, 40225 Düsseldorf, Germany; anand@hhu.de

**Keywords:** mitochondria, MICOS, cristae, cristae dynamics, cristae biogenesis

## Abstract

**Simple Summary:**

Mitochondria possess an outer and inner membrane. The part of the inner membrane parallel to the outer membrane is termed the inner boundary membrane, while the cristae membrane folds towards the mitochondrial matrix and houses the respiratory chain complexes. Crista junctions are located at the interface of the inner boundary membrane and the cristae membrane and contain the important ‘mitochondrial contact site and cristae organizing system’ complex. Despite the growing evidence that the mitochondrial inner membrane could remodel, cristae membranes were largely considered static for nearly seventy years, as the observations were mostly based on electron microscopy and tomography. Recently, using fluorescence super-resolution techniques, several studies showed that cristae membranes undergo dynamic remodeling in living cells, and probably even fission and fusion of the inner membrane. In this review, we discuss the important recent literature conveying the emerging role of the MICOS complex in cristae dynamics and its relation to cristae biogenesis. As the aberrant inner membrane architecture is connected to various pathologies such as cardiomyopathies, neurodegeneration and diabetes, understanding the roles of various molecules connected with cristae biogenesis and dynamics would shed light on the pathophysiology, probably leading to therapeutics in the near future.

**Abstract:**

Mitochondria are double membrane-enclosed organelles performing important cellular and metabolic functions such as ATP generation, heme biogenesis, apoptosis, ROS production and calcium buffering. The mitochondrial inner membrane (IM) is folded into cristae membranes (CMs) of variable shapes using molecular players including the ‘mitochondrial contact site and cristae organizing system’ (MICOS) complex, the dynamin-like GTPase OPA1, the F_1_F_O_ ATP synthase and cardiolipin. Aberrant cristae structures are associated with different disorders such as diabetes, neurodegeneration, cancer and hepato-encephalopathy. In this review, we provide an updated view on cristae biogenesis by focusing on novel roles of the MICOS complex in cristae dynamics and shaping of cristae. For over seven decades, cristae were considered as static structures. It was recently shown that cristae constantly undergo rapid dynamic remodeling events. Several studies have re-oriented our perception on the dynamic internal ambience of mitochondrial compartments. In addition, we discuss the recent literature which sheds light on the still poorly understood aspect of cristae biogenesis, focusing on the role of MICOS and its subunits. Overall, we provide an integrated and updated view on the relation between the biogenesis of cristae and the novel aspect of cristae dynamics.

## 1. Introduction: The Organization of the Mitochondrial Inner Membrane

**Figure 1:**
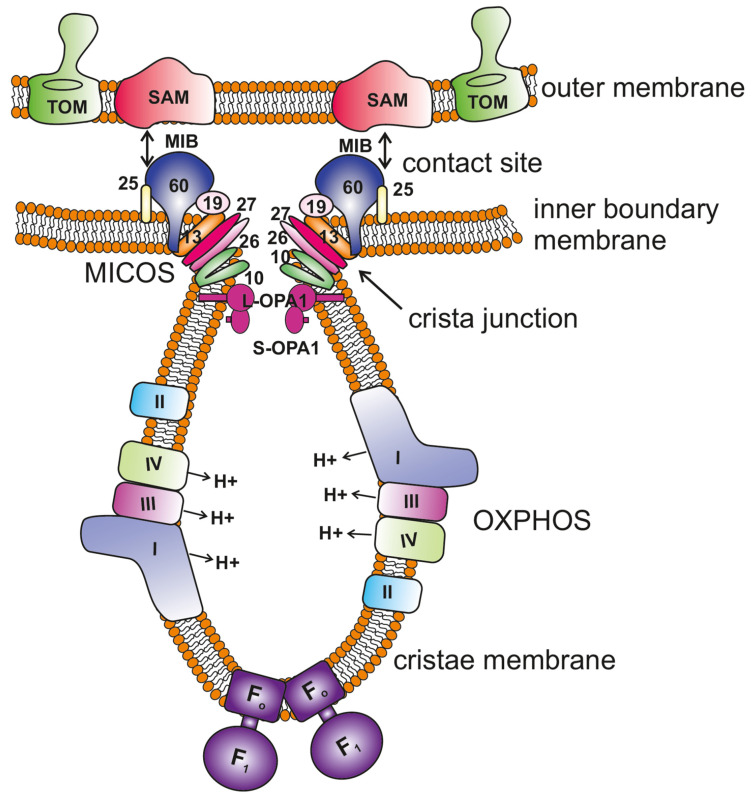
Key regulators of mitochondrial cristae organization. The scheme shows the organization of mitochondrial membranes where the cristae are formed by invagination of the inner membrane towards the matrix. The MICOS (mitochondrial contact site and cristae organizing system) complex resides at the crista junctions (CJs) and is composed of seven subunits, MIC10, MIC13, MIC19, MIC25, MIC26, MIC27 and MIC60 (only the numbers are depicted in the figure for the ease of legibility). MICOS is required to stabilize the CJs and form the contacts between the inner and outer membranes via interaction with the SAM (sorting and assembly machinery) complex. This interaction between MICOS and the SAM complex forms the larger complex called the mitochondrial intermembrane space bridging complex (MIB) that encompasses the intermembrane space. OPA1 is also enriched at the CJs, and interaction between membrane-bound long (L-) forms and soluble short (S-) forms is required to maintain the width of the CJs. F_1_F_O_ ATP synthase plays an important role in the formation of positive membrane curvature at the tip/rim of cristae. The OXPHOS (oxidative phosphorylation) machinery resides in the cristae membrane.

Mitochondria are important organelles that perform vital functions including energy conversion, cellular metabolism, apoptosis, calcium buffering and iron–sulfur cluster biogenesis. The mitochondrial ultrastructure is very versatile and thought to be optimally designed to perform many known mitochondrial functions. The hallmark feature of double membrane-enclosed mitochondria is the invaginations of the inner membrane (IM) into the matrix termed cristae. The rest of the IM that runs parallel to the outer membrane (OM) is termed the inner boundary membrane (IBM), which is compositionally and functionally distinct from the cristae membrane (CM) [1,2]. The CM and IBM are connected by small pore-like openings termed crista junctions (CJs) [3]. CJs restrict the entry of metabolites into the lumen of cristae due to their small diameter of 12–40 nm and hence potentially regulate many mitochondrial functions [4]. Cristae can vary in their shape, size and packing density [5] and undergo constant remodeling to adapt to varying energy demands and physiological cues [4,6,7,8,9,10]. Cristae are important identities containing the respiratory chain complexes (RCS) where oxidative phosphorylation (OXPHOS) occurs [1,2,11]. Altered cristae structures are found in many human diseases such as Parkinson’s disease, diabetes and cancer [12]. Although the ultrastructural features of cristae and CJs were described long ago [13], the fundamental question of how they are formed is not yet fully understood. The formation of cristae must be envisaged as a complex process involving a coordinated interaction of proteins and lipids helping to establish the curved nature of the CM and CJs. The latter display an approximately 90° bend in the membrane with both the leaflets exhibiting significant positive or negative curvature. Additionally, the rim/tip of the crista that seals the crista lumen from the matrix shows strong membrane curvature [14,15]. A team of various scaffolding protein complexes and phospholipid (PL) moieties could be envisioned to sculpt these membrane structures. Multiple models to explain cristae formation have been proposed [5] but have not been duly tested due to the technical challenges associated with addressing this fundamental question. The dynamin-like GTPase OPA1, the F_1_F_O_ ATP synthase complex and the MICOS complex are the three main known mediators of cristae formation thus far (Figure 1) [16].

### 1.1. The MICOS Complex Plays a Primary Role in IM Organization

Mic60 (initially termed ‘Formation of crista junction protein 1′ or, for short, ‘Fcj1′ in baker’s yeast) was shown to be located preferentially at CJs, to be part of a high-molecular weight membrane protein complex and to be mechanistically linked to CJ formation, in part by modulating the oligomeric state of the F_1_F_O_ ATP synthase in baker’s yeast [17]. For the mammalian MIC60 (termed Mitofilin, HMP or IMMT), it was shown earlier that Mic60 is part of a high-molecular weight complex enriched at the inner boundary membrane and that Mic60 is required for the formation of CJs [18]. Thus, Mic60 was the first component shown to be a central and evolutionarily conserved player in the formation of CJs that is located specifically at CJs. Later, the MICOS (mitochondrial contact site and cristae organizing system) complex consisting of Mic60 and several other subunits was discovered by three independent groups [19,20,21]. The MICOS complex is important for maintaining the cristae and CJ architecture and also mediates contact sites between the IM and OM. As we know now, it is overall highly conserved [22,23] and composed of seven subunits including MIC10/Minos1, MIC26/APOO, MIC27/APOOL, MIC13/QIL1, MIC19/CHCHD3, MIC25/CHCHD6 and MIC60/IMMT (Figure 1). It is largely enriched at CJs, and the deletion of subunits of the MICOS complex causes loss of CJs, leading to aberrant cristae structures that are detached from the IBM, resulting in the formation of stacks or concentric circles in the matrix [17,18,19,20,21]. The extent of the severity of the cristae phenotype varies with the deletion of individual subunits. MIC60 and MIC10 depletion shows the most severe defects with, virtually, a complete loss of CJs [24,25]. They were considered as key components of the MICOS complex and are most extensively studied. All the subunits of the MICOS complex with different structures and functions should act in unison to carry out the collective role of the MICOS complex in the formation of cristae structures. MIC26 and MIC27 belong to the family of apolipoproteins and cooperate with each other to regulate cristae structure. Therefore, while the single knockouts (KOs) show a mild phenotype, the double KO (DKO) leads to severe cristae defects [26]. MIC19 and MIC25 belong to the CHCHD (Coiled-Coil-Helix-Coiled-Coil-Helix domain) family of proteins and play a crucial role in the assembly and stability of the MICOS complex [27,28]. MIC13 is a small protein that shows no structural or functional homology to any known protein thus far. The MICOS subunits are organized into two subcomplexes: MIC60 subcomplex (MIC60–MIC19–MIC25) and MIC10 subcomplex (MIC13–MIC10–MIC26–MIC27). The two subcomplexes of MICOS are bridged together by MIC13, where loss of MIC13 causes subsequent depletion of subunits of the MIC10 subcomplex with the MIC60 subcomplex remaining intact [29,30,31]. One study in yeast indicated that these two subcomplexes of MICOS assemble independently in the proximity of the endoplasmic reticulum (ER)–mitochondria contact sites [32]. The MIC60 subcomplex can form a stable self-assembled subcomplex, whereas the MIC10 subcomplex assembly requires the activity of the ERMES (ER–mitochondria encounter structure) complex. It is worth exploring how the ERMES complex, which can have a plethora of functions, affects the formation of the MIC10 subcomplex. The question remains as to whether it is a direct role of the ERMES complex or an indirect effect due to changes in lipid composition. Mic27 is a lipid-binding protein and can perhaps be altered by the changes in PL environments. MICOS is further shown to interact with the sorting and assembly machinery (SAM) complex present in the OM to form a larger MICOS–SAM complex called the mitochondrial intermembrane space bridging (MIB) complex that traverses the intermembrane space (IMS) [23,33,34]. The SAM complex contains Sam50 and Metaxins 1, 2 and 3. The MIB complex is crucial in cristae formation as disruption in the MICOS–SAM axis also causes cristae defects, with loss of CJs [35]. An increasing number of interacting partners of MICOS subunits are being found, and there has been a debate over whether some of them belong to the MICOS complex or act as auxiliary components. The list includes proteins such as CHCHD10 [36], CHCHD2 [37], DNAJC11 [38], ARMC1 [39], DISC1 [40], VDAC, SLC25A46 [41], OPA1 [28], PINK1 [42], Miro [43], TFAM [44] and OMA1 [45]. The variety of functions that MICOS could perform, apart from cristae biogenesis, could be attributed to its interaction with the proteins mentioned. These include protein import, apoptosis, mitophagy, mitochondrial DNA organization, translation, transport and PL biogenesis [46,47]. Owing to its importance, alterations in the levels or modifications of MICOS subunits are found in many pathologies such as cancer, diabetes, neurodegenerative and cardiac diseases [12,48,49]. Mutations in genes of the MICOS complex, *MIC13*, *MIC26* and *MIC60*, are found in infantile lethal mitochondrial hepato-encephalopathy [50,51,52], mitochondrial myopathy associated with cognitive impairment [53] and Parkinson’s disease [42], respectively.

### 1.2. Other Important Molecular Players Playing Vital Roles in IM Organization

OPA1 plays a dual role in mitochondrial fusion and cristae biogenesis [8]. The regulation of OPA1 is highly complex as it contains eight splice variants and two proteolytic cleavage sites that can collectively give rise to long and short forms of OPA1 [54,55,56]. The complex between the long and short OPA1 forms is proposed to maintain the width of CJs, which can restrict the movement of metabolites such as cytochrome *c*, a proapoptotic factor, inside the cristae lumen [57,58,59,60]. During apoptosis, the OPA1 complex is disassembled which causes widening of CJs and subsequent release of cytochrome *c* to initiate the apoptotic cascade [57,61]. Depletion of OPA1 leads to a reduced number of cristae that are usually swollen along with an increase in the width of CJs. Still, CJs can be formed in the absence of OPA1 in mammalian cells [62]. The F_1_F_O_ ATP synthase, well known for its role in the synthesis of ATP, plays an important role in the formation of the membrane curvature at the rim/tip of the cristae via its intrinsic ability to form dimers and oligomers. The dimers of the F_1_F_O_ ATP synthase complex are formed by interactions between the adjacent membrane-bound F_O_ domain at a defined angle that cause bending of the CM and thereby generate a strong positive curvature at cristae tips/rims [63,64,65].

## 2. Understanding Cristae Architecture Using Recent Technological Advancements

In recent years, many technical advancements that include live-cell imaging using super-resolution (SR) nanoscopy, focused ion beam-scanning electron microscopy (FIB-SEM), electron tomography (ET), single-particle tracking (SPT) and fluorescence recovery after photobleaching (FRAP) have been used to address the fundamental questions about cristae biogenesis and dynamics [24,25,66,67,68]. These techniques have allowed unexpected insights into the dynamic nature of cristae and have changed our view of cristae being static entities that only display different shapes under varying circumstances. An important change in perception occurred with the discovery that cristae can undergo dynamic membrane remodeling at a timescale of seconds in a MICOS-dependent manner [68]. The MICOS subunit MIC13 was identified as a central regulator in these processes in mammalian cells. In this section, we discuss the recent advancements in cristae dynamics and biogenesis with a focus on the emerging role of the MICOS complex in regulating these processes.

### 2.1. Application of Fluorescence Super-Resolution Techniques to IM Reveal Novel Insights

SR techniques provide crucial insights into the organization and distribution of the MICOS complex in the IM, showing a typical rail-like arrangement of MIC60 and other MICOS subunits across the mitochondrial length, mimicking the arrangement of CJs in electron micrographs [24,26,69,70]. Specifically, dual staining of MIC60 and cristae showed that, as expected for a CJ protein, most of MIC60 is found associated with cristae except a few instances where MIC60 spots were found in the IBM [25]. The two-sided distribution of MIC60 on either side of the mitochondrial length and its colocalization with OM protein remained unaltered in the *MIC10* KO, showing the important role of MIC60 in marking the sites for nascent CJs and contact sites [24,70,71]. The arrangement of MIC60 within an individual CJ was identified using the powerful MINFLUX nanoscopy technique that could attain a resolution of around 5 nm by employing the combinational advantages of photo-activated localization microscopy (PALM), stochastic optical reconstruction microscopy (STORM) and stimulated emission depletion (STED) microscopy [72]. Multiple MIC60 molecules were arranged in a ring-like pattern with a diameter of around 40–50 nm that could surround a CJ [72]. MIC19 was in close proximity of MIC60 compared to MIC10.

### 2.2. MICOS Complex Regulates Apparent Cristae Fusion and Fission Cycles

**Figure 2:**
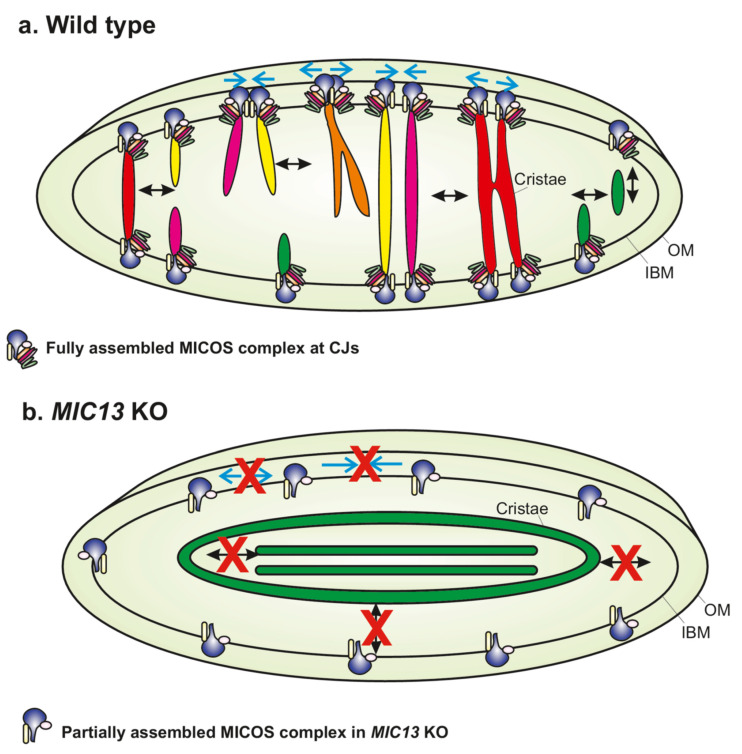
The MICOS complex regulates apparent cristae fusion and fission cycles. (**a**) Scheme depicts the various kinds of crista and CJ dynamics that are found in a mammalian cell. CJs formed by the MICOS complex move towards and away from each other in order to undergo merging and splitting events, respectively, as shown by cyan arrows. Similarly, cristae show a dynamic movement that involves continuous fusion and fission cycles in a balanced and reversible manner. In various instances, merging CJs can bring with them the adjoining cristae and facilitate their fusion along their length, resulting in the formation of a cristae network that resembles the ‘X’ or ‘Y’ letter. Cristae fusion is immediately followed by fission or vice versa. Cristae can also detach from the inner boundary membrane (IBM) to form cristae vesicles (shown as green cristae). These cristae vesicles can re-fuse to the IBM. OM represents outer membrane. (**b**) In *MIC13* KO, cristae are arranged as concentric rings or internal stacks that are not connected to the IBM. The movement of CJs and cristae is drastically reduced in *MIC13* KO cells.

Recent studies using SR techniques showed unexcepted dynamics of cristae [24,66,68,73,74,75]. Both cristae and CJs constantly changed their position within mitochondria, confirming that they are highly dynamic within seconds [24]. Tracking movements of CJs using live-cell STED nanoscopy showed that they repeatedly come together and then move apart in a balanced and reversible manner. Interestingly, two CJs coming together bring with them the adjoining cristae so that the cristae appear as the letter ‘Y’, as visualized using a time-lapse movie of mitochondria expressing MIC13-SNAP, which dually marked cristae and CJs (Figure 2). Likewise, cristae merge along the length of cristae (X- or Y-type merging) or with the IBM. On several occasions, the cristae merging events were followed by subsequent splitting events either at or in the vicinity of previous merging events in a ‘kiss-and-run’ manner. These results were corroborated by another study that also found a similar pattern of cristae movement [66]. While it should be kept in mind that one cannot distinguish two adjacent cristae and CJs which are closer than 50 nm, the resolution attained with live-cell STED nanoscopy [24], there were instances where contacts between cristae coincided with the immediate re-distribution of TMRM, a membrane potential-sensitive dye, from one crista to another, demonstrating mixing of content between the cristae during merging events. The content mixing between the cristae was corroborated using techniques involving photoactivable ATP5I-GFP and PEG-based fusion between mitochondria harboring differently labeled ATP5I probes targeted to the cristae [24]. This led to the proposal of the ‘Cristae Fission and Fusion’ (CriFF) model, where cristae undergo continuous cycles of fusion and fission, often assisted by the dynamics of CJs [24]. Interestingly, deletion of *MIC13*, impairing, e.g., MICOS assembly, leads to a drastic reduction in merging and splitting events of cristae and CJs, identifying MICOS as the first molecular player that is required for the dynamics of cristae and CJs (Figure 2). It is therefore possible that the holo–MICOS complex is required for efficient cristae dynamics where it functions to coordinate the movement of a certain fraction of cristae towards and away from each other. Recently, another study found that loss of MIC10, MIC19, MIC60 and SAM50 caused a drastic reduction in various forms of cristae movement and dynamics, emphasizing the central role of MICOS in regulating cristae dynamics [66]. In addition, while the *OPA1* KO showed a drastic reduction in cristae dynamics, the *YME1L* KO and *ATAD3* KO showed an increase in cristae dynamics [66]. YME1L is an IM protease that regulates, among other substrates, MIC60 processing [76]. ATAD3 has been shown to progressively affect cristae morphology in a mouse muscle-specific KO [77] and was identified as an interacting factor of the MICOS complex [66]. The question remains how the MICOS complex and these proteins regulate cristae dynamics. Though further experiments are required to understand the relationship between the MICOS complex and cristae dynamics, the fact that cristae and CJ dynamics occur in a similar reversible and balanced manner in comparable timescales would suggest that they are mechanically linked. One possibility could be that the MICOS complex forms stable CJs that act as an anchor to facilitate the cristae fusion and fission, for example, in Y-type merging events. In fact, *ATAD3* KO and *YME1L* KO cells, which contain a higher number of CJs compared to control mitochondria, also display an increase in cristae dynamics [66], indicating a positive correlation between the number of CJs and cristae dynamics. Another possibility, which is not mutually exclusive, is that the MICOS complex dynamically forms CJs which could lead to the formation of transient isolated cristae vesicles by membrane fission and re-fusion to the IBM or adjacent cristae. This is supported by the fact that stacks of cristae are found in the matrix in most of the KOs of the MICOS subunits. These possibilities do not exclude that MICOS regulates cristae dynamics independent of its function in the formation of CJs. Some of the cristae fusion events that occur along the length of the cristae could be independent of the presence of CJs.

### 2.3. Possible Implications of Cristae Dynamics

As the field of cristae dynamics is still in infancy, future experiments could provide better insights about the consequences of the reduced cristae or CJ dynamics in the KOs of the MICOS subunits and their relevance in many pathologies associated with the MICOS complex. Cristae dynamics may serve vital functions in mitochondria at different levels. Cristae housing OXPHOS complexes might, for example, employ cristae dynamics to dilute the local accumulation of dysfunctional OXPHOS complexes by mixing with optimally functioning complexes or subunits representing an efficient intramembrane complementation system. In addition, cristae dynamics may help to dynamically regulate the accessible surface of the IM and, conversely, the ability to trap protons efficiently. The transient formation of cristae vesicles could control the volume of the intermembrane space and the intracristal space, containing various metabolites, and thereby regulate their exchange with the cytosol. Protons as well as pools of ATP or other metabolites could be trapped efficiently in this way. Conversely, by fusion of cristae to the IBM, a larger ‘exchange-capable’ IM surface is generated. Overall, this could help to make oxidative phosphorylation more efficient and, importantly, presumably more tunable within a short timescale. An overview of the various possible implications of cristae dynamics has been discussed in more detail before [68].

### 2.4. MICOS Defines Heterogeneity in Membrane Potential within Individual Mitochondria

Cable theory considered mitochondria as an electric cable with a homogenous voltage across the entire length of a particular mitochondrion due to the reason that disruption of the voltage in a portion of the mitochondrion perturbed the voltage across the entire mitochondrion [78,79]. However, these observations were primarily limited by technological developments at that time. The high-resolution Airyscan-based approach and STED nanoscopy that could resolve cristae and the IBM showed that mitochondrial membrane potential (ΔΨ_m_)-sensitive dyes (such as TMRE) were largely sequestrated into cristae [67]. Moreover, the level of TMRE associated with cristae was found to be higher than in the IBM, indicating that the ΔΨ_m_ was significantly higher at the CM compared to the IBM. Further, and possibly even more striking, there was the observation that there was extensive heterogeneity in the ΔΨ_m_ of individual cristae within a mitochondrion. This was not compatible with cable theory and asked for a novel concept. When a segment of mitochondria was depolarized using laser induction and imaged using high-resolution techniques coupled with fast imaging, it could be observed that local segments were depolarized [67]. The difference in the ΔΨ_m_ between the IBM and CM could be increased and decreased by the addition of oligomycin, an ATP synthase inhibitor, and CCCP, a protonophore, respectively. Interestingly, the KO of MICOS subunits *MIC13*, *MIC60* or *MIC10* showed a reduction in the heterogeneity along the IM and a decrease in the difference in the ΔΨ_m_ between the CM and IBM [67]. This clearly indicates a critical role of CJs in maintaining this heterogeneity perhaps by acting as a diffusion barrier for metabolites or protons. In line with this, cristae vesicles arising due to detachment of cristae from the IBM in the *MIC10* KO (or the DKO of *OPA1* and *DRP1*) were hyperpolarized [67]. The *OPA1* KO also showed a similar reduction in the intramitochondrial ΔΨ_m_ heterogeneity. Overall, these findings indicate that proper crista and CJ organization is essential for maintaining the heterogeneity of the membrane potential in the IM. It is also worth pondering why individual cristae display extensive heterogeneity in the ΔΨ_m_ within a mitochondrion despite apparent cristae fusion and fission events. Perhaps, the generation of heterogeneity in the ΔΨ_m_ at the level of individual cristae operates similar to the way it does at the level of individual mitochondria. While mitochondria undergo cycles of fission and fusion, fission events resulted in the generation of daughter mitochondria, with one having a much higher ΔΨ_m_ than the other [80]. We speculate that such events also occur at the intramitochondrial level, resulting in different cristae possessing dissimilar ΔΨ_m_. In certain cases, the difference in ΔΨ_m_ could occur due to the age of individual cristae, position of cristae within the mitochondria and/or density of RCS within individual cristae. This probably points to intramitochondrial quality control mechanisms that are yet to be discovered.

## 3. Role of MICOS Complex in Regulating Cristae Biogenesis

### 3.1. Evolutionarily Conserved MIC60 and MIC10 Can Bend Membranes to Form CJs

**Figure 3:**
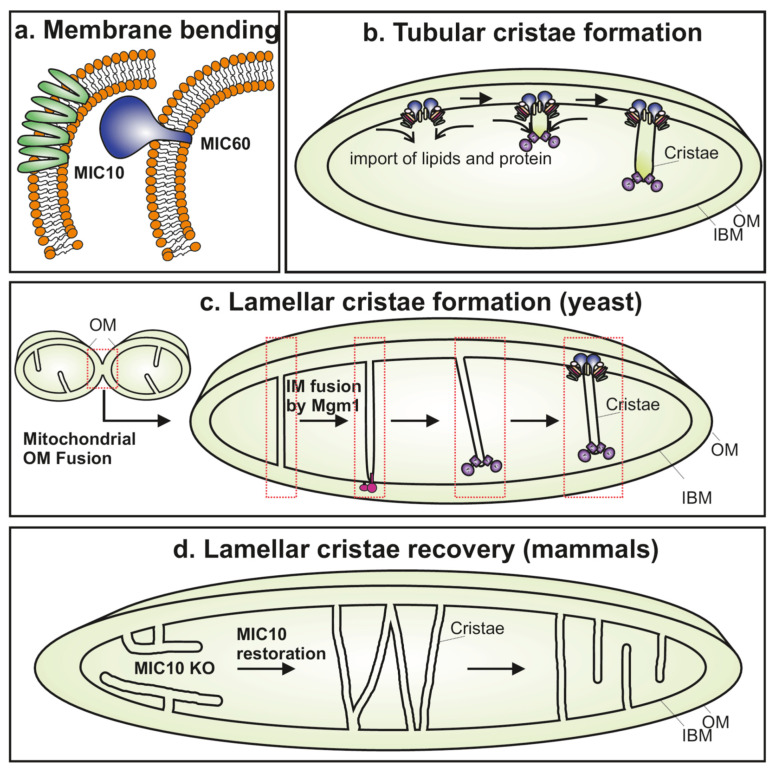
Various roles of the MICOS complex in regulating cristae biogenesis. (**a**) MIC10 and MIC60 can generate membrane curvature by bending the lipid bilayer. Mic10 has a hairpin topology and the oligomerization of Mic10 results in membrane bending. Mic60 contains an amphipathic helix that is known to generate membrane curvature important for membrane bending activity. (**b**) The MICOS complex is shown to initiate the formation of tubular cristae in yeast that involves the influx of lipids and proteins as the primary steps. F_1_F_O_ ATP synthase oligomers at cristae tips/rims are also depicted. (**c**) The lamellar cristae in yeast are proposed to be formed during the process of inner membrane fusion by Mgm-1 (OPA1 homolog). MICOS is required to complete the formation of these lamellar cristae by formation of CJs involving the assembly of the MICOS complex. (**d**) Mammalian *MIC10* KO cells contain few tube-like cristae, and the following re-synthesis of MIC10 over time leads to a recovery of lamellar cristae. This is proposed to mainly occur via restructuring of existing unstructured tubes to form lamellar cristae rather than de novo cristae biogenesis.

MIC60 is the most ancient subunit of the MICOS complex as it could be traced back to alpha-proteobacteria, which are bacterial progenitors of mitochondria even containing rudimentary cristae called intracytoplasmic membranes (ICM), pointing to the endosymbiotic origin of mitochondrial cristae [22,23,81]. MIC60 and MIC10 were most widespread and found in every major lineage of eukaryotes, except in some taxa that lack mitochondrial cristae, suggesting an evolutionarily conserved role of MICOS in cristae formation. In line with this, the expression of the IMS domain of Mic60 in *Escherichia coli* showed formation of the ICM, and, conversely, the alpha-proteobacteria Mic60 was able to partially rescue the morphology of mitochondria and cristae as well as respiratory defects of Mic60-deficient baker’s yeast [82]. How do MIC60 and MIC10 form a cristae structure? Using in vitro reconstitution assays, it was shown that both Mic60 and Mic10 have the capacity to bend membranes (Figure 3a) [82,83,84]. Purified Mic60 deformed liposomes and formed long, branched, tubular structures with a diameter similar to cristae [82,84]. Mic60 consists of a mitochondrial targeting sequence and a transmembrane domain on the N-terminus, whereas the remaining portion exposed to the IMS consists of a central coiled-coil domain and a conserved C-terminal mitofilin signature domain [85,86]. An amphipathic helix identified between the coiled-coil domain and mitofilin domain was crucial for the membrane bending activity of Mic60 [84]. Amphipathic helices are well-known mediators for sensing or creating positive membrane curvature [87]. Deletion of the conserved amphipathic helix region in thermophilic fungus *Chaetomium thermophilum* or presence of point mutations in key conserved residues in this helix, for instance, Arg572 and/or Phe573, prevented lipid binding and tubulation of liposomes and failed to restore the mitochondrial cristae defects of Mic60-deficient yeast [84], indicating the requirement of the membrane binding activity of Mic60 for the maintenance of cristae structure. While the mitofilin domain of Mic60 negatively regulates the membrane bending activity, binding of Mic19 to the mitofilin domain could alleviate this negative effect and enhance the membrane binding and tubulation of liposomes via Mic60 [84]. Interestingly, yeast Mic19 showed two redox forms, and only the oxidized form was bound to Mic60 [88]. Mic19 was suggested as a redox-dependent regulator of MICOS as oxidation of Mic19 was important for the stability of the whole MICOS complex [88], probably because the oxidized form of Mic19 acts as a connector between two MICOS subcomplexes [71].

Mic10 can also bend membranes, albeit using a different mechanism involving its homo-oligomerization [83,89,90]. Mic10 acquires a hairpin-like topology as it contains two transmembrane (TM) domains that span the IM twice so that both the N- and C-terminus face the IMS. Indeed, the purified Mic10 could deform liposomes such that they form tubular structures of a very small diameter resembling cristae [83]. The conserved GxGxGxG motifs in both the TM regions were crucial for the formation of the Mic10 oligomers as well as liposome deformation [83,89]. It is proposed that the oligomerization of Mic10 via its GxGxGxG motifs results in membrane bending. GxGxGxG in Mic10 consists of two entwined GxxxG motifs [83,89]. GxxxG motifs are found in the TM domain of many proteins and are known to be crucial for TM oligomerization and helix-helix packing [91]. The GxxxG motif in subunit *e* or *g* is crucial for the formation of oligomers of the F_1_F_O_ ATP synthase complex, which are required for the formation of cristae rims/tips [92,93]. Expression of the Mic10 variants with mutations in the conserved glycine residues failed to rescue the cristae defects found in an Mic10-deficient yeast strain, indicating the important role of this motif in cristae structure [83,89]. Overexpression of Mic10 in yeast could form Mic10 oligomers independent of other MICOS subunits and led to highly interconnected cristae with multiple slit-like and branched CJs [89]. On the other hand, Mic60 overexpression showed an increased number and branching of cristae [17], a phenotype distinct from Mic10 overexpression, which could be attributed to the differences in their mechanism of action to deform the membrane.

### 3.2. Differential Role of MICOS Complex in Formation of Lamellar and Tubular Cristae

Even though MIC10 and MIC60 can bend membranes and this might be sufficient for the formation of the ICM in prototype organisms, the cristae structure in higher organisms is very complex and would be formed via complicated mechanisms requiring the interplay between various scaffolding proteins and lipid assemblies. A variety of cristae shapes exist depending upon the cell type and function [5]. Lamellar cristae are usually found in tissues with high energy demands, perhaps due to the large planar area that can accommodate more OXPHOS complexes [47]. Tubular-shaped cristae are found, for example, in tissues specialized for lipid metabolism such as steroidogenic tissues [5,47]. New models proposed for cristae biogenesis based on recent insights show a dominant role of MICOS in sculpting cristae structure. In baker’s yeast, Walter Neupert and colleagues suggested that while the lamellar cristae are formed during mitochondrial IM fusion which occurs next to OM fusion, the tubular cristae are formed by invagination of a growing IM due to influx of proteins and lipids (Figure 3b,c) [94]. After the fusion of OM, Mgm1 (human homolog of OPA1) initiates fusion of the two opposing IMs which fuse at one point and continue around their edges, resulting in the formation of a sac-like nascent lamellar crista (Figure 3c). Though MICOS was not required in the initial step of formation of the lamellar cristae, the process was terminated by assembly of the MICOS complex which serves to form stable CJs (Figure 3c). Assemblies of F_1_F_O_ ATP synthase oligomers help to form and stabilize the tips or rims of cristae (Figure 3c) [17,64]. This is in line with the observation that MICOS-deficient cells contain stacks that have a composition of proteins similar to cristae [94], and that cells not able to form F_1_F_O_ ATP synthase oligomers show interconnected cristae with CJs [17]. On the contrary, MICOS assembly was proposed to initiate the formation of tubular cristae (Figure 3b). The existence of these two pathways was supported by another study that showed double depletion of Mgm1 and Mic60 causes a severe decrease in cristae structures, suggesting parallel roles of Mgm1 and Mic60 in different pathways of cristae formation [95]. Similar defects were seen in strains that were simultaneously deleted for *Mgm1* and *Mdm35*, the latter being an important component of the Ups–Mdm35 complex that is required for PL transport in mitochondria [95]. This suggest that MICOS and PL import are cooperatively required for the formation of tubular cristae. Most of these studies were conducted in yeast, and it is not clear if these pathways are conserved in higher eukaryotes. The absence of either mitochondrial fusion or fission in human cells still allowed the formation of lamellar cristae, indicating that these processes are not necessary for the formation of lamellar cristae in human cells [25]. On the contrary, in yeast, the absence of mitochondrial fusion and/or fission resulted in a loss of lamellar cristae [94], indicating differences in yeast and mammalian mitochondria. Although the fusion and fission machinery are largely conserved from yeast to human, perhaps the process of cristae formation is, in part, regulated differently in humans compared to yeast. The *OPA1* KO showed a minor reduction of 10% in the number of lamellar cristae, indicating that perhaps only a fraction of lamellar cristae could be formed by IM fusion in higher organisms [25]. Experiments involving tracking of mitochondrial cristae formation using SR nanoscopy immediately after mitochondrial fusion in mammalian cells could provide valuable insights into this process [66]. A recent study indicated an important role of MICOS in the formation of lamellar cristae in mammalian cells (Figure 3d) [25]. Using a combination of state-of-the-art technologies such as FIB-SEM, 3D ET, STED and MINFLUX nanoscopy, it was shown that MIC60 and MIC10 are each specifically required for the formation of CJs and lamellar cristae, respectively [25]. While the *MIC60* KO virtually lacked CJs, the *MIC10* KO still contained few tubular CJs with a larger diameter, suggesting that the MIC60 subcomplex is required for the formation of CJs. On the contrary, *MIC10* KO cells contained only large perforated tube-like cristae. This shape of cristae resulted in no changes in the two-sided distribution of MIC60 in the IBM on either side of mitochondria in the *MIC10* KO when compared to control cells; however, the distribution of MIC60 which exhibited a stripe-like pattern resembling cristae in WT was absent in the *MIC10* KO. The formation of cristae and CJs was studied using an inducible expression system, and the recovery of cristae morphology was tracked upon re-introduction of MIC60 or MIC10 in the respective KO [25]. It was proposed that there was no cristae biogenesis but rather remodeling of existing cristae. In the case of re-introduction of MIC60 in the *MIC60* KO, an intermediate cristae morphology was found that contains CJs appearing on aberrant crista, termed as secondary CJs [25]. Re-introduction of MIC10 in the corresponding KO led to the formation of lamellar cristae containing tube-like cristae, suggesting that the formation of the holo–MICOS complex leads to its spatial redistribution and subsequent formation of lamellar cristae from tube-like cristae in the *MIC10* KO (Figure 3d). While this study provided insights about how the cristae structure is re-established in MICOS KOs, the generation of nascent lamellar cristae during normal physiological conditions may still be differently regulated. In this context, MICOS-dependent cristae fusion and fission cycles could be directly related to cristae biogenesis: fission of cristae from the IBM could generate a cristae vesicle and vice versa, and cristae fusion events can contribute to cristae biogenesis [24,66,68]. Future experiments are required to determine the link between cristae dynamics and cristae biogenesis via the MICOS complex and other factors.

### 3.3. MICOS and Cardiolipin Influence Cristae Structure Formation

Cardiolipin (CL) is known as the signature lipid of mitochondria and, due to its conical shape, has been shown to play a vital role in cristae biogenesis [15]. Cardiolipin together with another non-bilayer PL, phosphatidylethanolamine (PE), constitutes 50 % of IM PLs and mainly segregates towards the negative curvature of the membrane to stabilize cristae folds. There is ample evidence suggesting a direct or indirect regulation of CL by the MICOS complex and vice versa. Depletion of apolipoproteins of the MICOS complex, MIC26 and MIC27, leads to decreased levels of tafazzin [96], and MIC27 binds to CL [97]. Tafazzin is the transacylase enzyme that is required for the maturation of CL. Mutations in the X-linked taffazzin gene are linked to Barth syndrome. MIC26 and MIC27 reciprocally regulate each other such that the protein level of one is increased upon downregulation of the other one and vice versa [96,98]. The DKO of MIC26 and MIC27 showed a decrease in CL levels, while the composition of the acyl chains was not perturbed [26]. In addition, the DKO showed a decrease in respiration and reduced stability and integrity of respiratory chain (super) complexes and F_1_F_O_ ATP synthase. Interestingly, overexpression of cardiolipin synthase could rescue these respiratory defects, suggesting an important role of CL in mediating these defects [26]. It is not yet clear how MIC26 and MIC27 regulate CL levels. The observations that CL-synthesizing enzymes form a huge complex that interacts with MIC60 and MIC19 [99] and that the MICOS complex is perturbed in Barth syndrome patient mitochondria [100] indicate a possible role of the MICOS complex in regulating CL organization and vice versa. This is supported by many lines of evidence in baker’s yeast, where a negative genetic interaction was shown between the MICOS complex and CL-synthesizing enzymes [20]. Moreover, CL was required to stabilize the Mic10 oligomers [98] and integration of Mic27 into the MICOS subcomplex [71]. The deletion of the auxiliary MICOS interactor Aim24 when combined with additional modification (His-tag to Mic12 or Mic27) could lead to an alteration in the acyl chain composition of CL [101]. The most striking correlation between cristae and CL levels was shown during simultaneous deletion of *Mgm1* and *Mic60* which led to a loss of cristae structure, accompanied by a dramatic decrease in CL levels [95]. The authors provide the following explanations to this observation: (i) cristae could act as a reservoir for CL, and a reduced number of cristae destabilized CL, or (ii) loss of cristae perturbed the CL synthesis as specialized regions in cristae might be required for transport and synthesis of PL. In any case, this points to a tight regulation between cristae structure and CL, which could be mediated largely by the MICOS complex. In line with the second scenario, a direct role of the MICOS complex and the formation of OM–IM contact sites for the generation of PE was shown in baker’s yeast [102]. Moreover, *Arabidopsis thaliana* Mic60 (AtMic60) was shown to play a role in trafficking PE [103]. Overall, there is an intricate relationship between MICOS, CL and lipid transport that awaits further discoveries.

## 4. Conclusions

The understanding of how cristae are formed and remodeled has progressed rapidly after the discovery and characterization of the first MICOS subunit about a decade ago. Several new findings have revealed that cristae are highly dynamic entities and that a central role regulating this can be attributed to the MICOS complex. These studies have opened up many new avenues to explore exciting questions pertaining to cristae remodeling during various physiological conditions. Cristae are highly dynamic and undergo apparent fusion and fission cycles at the timescale of seconds. The question remains as to how these fast cristae dynamics mediated by the MICOS complex influence cristae formation and biogenesis and vice versa. The precise function of cristae dynamics and the mechanisms of how this is brought about by the MICOS complex are also exciting to explore further. The MICOS complex has been increasingly associated with many pathological conditions and human diseases. The changes in cristae dynamics and biogenesis could influence the pathobiology of these diseases.

## Data Availability

Not applicable.
